# Artificial plateau construction during the Preclassic period at the Maya site of Ceibal, Guatemala

**DOI:** 10.1371/journal.pone.0221943

**Published:** 2019-08-30

**Authors:** Takeshi Inomata, Daniela Triadan, Flory Pinzón, Kazuo Aoyama

**Affiliations:** 1 School of Anthropology, University of Arizona, Tucson, Arizona, United States of America; 2 Facultad de Ciencias Sociales, Universidad del Valle de Guatemala, Guatemala City, Guatemala; 3 Faculty of Humanities, Ibaraki University, Mito, Ibaraki, Japan; University at Buffalo - The State University of New York, UNITED STATES

## Abstract

Investigations at the Maya site of Ceibal, Guatemala, documented an artificial plateau, measuring 600 x 340 m in horizontal dimensions and 6 to 15 m in height. Unlike highly visible pyramids, such horizontally extensive constructions covered by the rainforest are difficult to recognize on the ground, but airborne laser scanning (LiDAR) revealed its planned form. Excavations carried out over many years provided data on its construction sequence, fill volumes, and labor investments. The initial construction of the plateau occurred around 950 B.C. when a formal ceremonial complex was built in its center. This was the period when the inhabitants of the Maya lowlands were adopting a new way of life with greater reliance on maize agriculture, full sedentism, and ceramic use. The inhabitants of areas surrounding Ceibal, who retained certain levels of residential mobility, probably participated in the construction of the plateau. In this regard, the Ceibal plateau is comparable to monumental constructions that emerged before or during the transition to agriculture or sedentism in other parts of the world. The data from Ceibal compel researchers to examine the social implications of monumental constructions in the Maya lowlands before the establishment of centralized polities with hereditary rulers. Unlike pyramids, where access to the summits may have been limited to privileged individuals, the horizontal monumentality of the plateau was probably more conducive to inclusive interaction. The Ceibal plateau continued to be built up during the Preclassic period (1000 B.C.-A.D. 175), and its fill volume substantially surpassed those of pyramids. Large-scale construction projects likely promoted organizational and managerial innovations among participants, which may have set the stage for later administrative centralization.

## Introduction

Pyramidal buildings are commonly viewed as the hallmarks of Maya and other Mesoamerican civilizations. At the height of population and political centralization during the Classic period (A.D. 250–950), these buildings were foci of collective work at many Maya centers and represented the power and authority of rulers and other elites. Importantly, some of the largest known pyramids in the Maya lowlands were constructed before the Classic period. For example, the Danta pyramid complex of El Mirador, built during the Late and Terminal Preclassic period (350 B.C.-A.D. 250), measures 72 m in height, dwarfing any pyramids of later periods [1,2]. It is likely that the construction of such buildings played a critical role in the development of centralized polities in the region. Recent investigations, however, hint at the presence of monumental constructions that emphasized horizontal dimensions, as opposed to the vertical ones of pyramids. Such artificial plateaus may have been even more important than pyramids during the Middle Preclassic period (1000–350 B.C.), but they have received little attention from researchers. A notable exception is the site of Cival, where Estrada-Belli uncovered the large-scale leveling of a hilltop area of 0.5 km^2^ dating toward the end of the Middle Preclassic [3,4].

**Fig 1:**
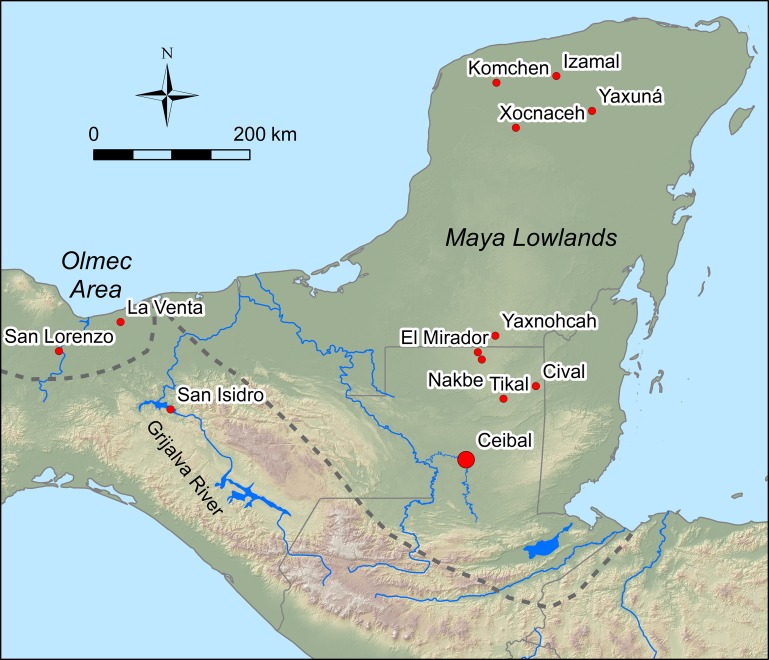
Map of the Maya area and adjacent regions with the locations of the sites discussed in the text.

Our airborne LiDAR (Light Detection and Ranging) survey and excavations at the site of Ceibal, Guatemala, also documented a large artificial plateau (Fig 1). Its initial version dates back to the beginning of the Middle Preclassic. Estimates of its fill volumes and construction costs indicate that the Ceibal plateau was a massive construction even at its early stages, achieved through significant labor investments by a large part of the population. Like the case of Cival, its volume significantly exceeds those of pyramids. The Ceibal plateau was among the earliest-documented monumental constructions in the Maya lowlands.

## Ceibal and its plateau

### General setting

**Fig 2:**
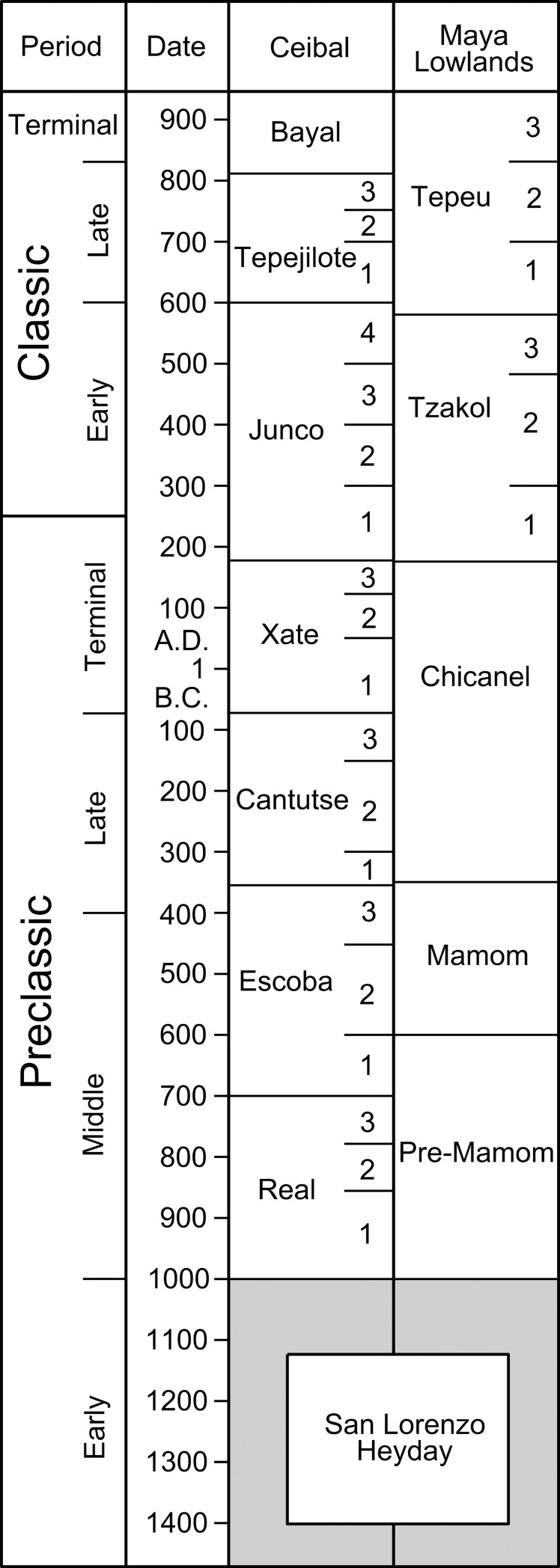
Chronology of Ceibal.

Ceibal is the largest Maya center in the southwestern part of the Maya lowlands, located on top of an escarpment overlooking the Pasión River (Fig 1). Willey directed the Harvard University Project (HP) at this site from 1964 through 1968 [5–7]. We revisited this important site in 2005 as the Ceibal-Petexbatun Archaeological Project (CPAP) and continued our field research until 2017. Permits for the CPAP research were granted by the Instituto de Antropología e Historia de Guatemala (IDAEH). Detailed stratigraphic and ceramic data, along with 182 radiocarbon dates obtained so far, allowed us to refine the chronology of Ceibal’s occupation, originally developed by Sabloff during HP (Fig 2)[6].

The ceramics of the Junco 1 phase generally align with those of the Terminal Preclassic in other parts of the Maya lowlands, and Junco 2–4 materials with those of the Early Classic. Nonetheless, the transition from Junco 1 to Junco 2 is not clear in some contexts. Thus, in the following analysis, we use the Xate phase as the end of the Preclassic period (A.D. 175), and the Junco 1 phase is included in the Early Classic period. For the discussion of the Maya lowlands in general, however, we use the more conventional date of A.D. 250 for the end of the Preclassic. The absolute dates are based on radiocarbon dates, which we analyzed with Bayesian statistics. All dates are calibrated. These radiocarbon dates and the Bayesian models are discussed in detail in our previous publications [8–10]. Since our last publication, we have obtained additional radiocarbon assays, resulting in a total of 182 dates. These recently obtained dates confirm our chronology and do not change the absolute dates of the ceramic phases published in our previous publications [11].

The residents of Ceibal began to use ceramics and to build durable structures at the beginning of the early Middle Preclassic Real phase around 1000 B.C. Ceibal continued to grow during the late Middle Preclassic Escoba phase (700–350 B.C.) and the Late Preclassic Cantutse phase (350–75 B.C.). After some domographic fluctuations during the Terminal Preclassic Xate phase (75 B.C.-A.D. 175), it experienced a significant demographic decline at the end of the Junco 1 phase around A.D. 300. The Early Classic Junco 2–4 phases (A.D. 300–600) are characterized by low populations and limited construction activity. Its population grew again during the Late Classic Tepejilote phase (A.D. 600–810). After a political disruption at the end of the Tepejilote phase, Ceibal regained political power during the Terminal Classic Bayal phase (A.D. 810–950) amid the so-called Classic Maya collapse. It was, however, deserted at the end of this period. During the Postclassic Samat phase (A.D. 1000–1200), small groups visited this place.

In 2015, we acquired airborne LiDAR data over an area of 470 km^2^ around Ceibal [12,13]. LiDAR involves laser scanning of terrain from equipment placed on a small airplane. Some laser pulses penetrate through the canopy and record the 3-dimensional morphology of the ground surface. In the tropical lowlands where thick vegetation hinders pedestrian surveys of wide areas, LiDAR has revolutionary, providing detailed data on subtle archaeological features over extensive areas [14–19]. The crew of the National Center for Airborne Laser Mapping (NCALM) of the University of Houston, under the direction of Ramesh Shrestha and the coordination of Juan Carlos Fernandez Diaz, obtained LiDAR data over the area of Ceibal from March 18th to 23rd, 2015. They collected data for most parts of the study area from a flying altitude of 700 m above the ground level (AGL) and at a total pulse repetition frequency (PRF) of 450 kHz (150 kHz per channel for the three channels of Titan LiDAR), but they also used a total PRF of 750 kHz for some flight lines. For the central part of Ceibal, the team conducted canopy penetration tests with multiple settings, including 700 m AGL and 300 kHz total PRF, 600 m and 450 kHz, and 400 m and 150 kHz. The canopy penetration test flights resulted in 51 to 72 laser shots per m^2^, whereas regular mapping flight lines produced 15 to 19 shots per m^2^. Ground point densities vary widely by vegetation type, but the average for the central part of Ceibal obtained by the test flights, where the plateau is located, was 2.84 points/m^2^. The NCALM team then produced a digital elevation model (DEM, bare earth model after the removal of vegetation and buildings) at a horizontal resolution of 0.5 m. For a more detailed discussion of the LiDAR acquisition methods and settings at Ceibal, see [12,13,20]. Other applications of LiDAR in tropical areas include [14–16,19,21–25].

**Fig 3:**
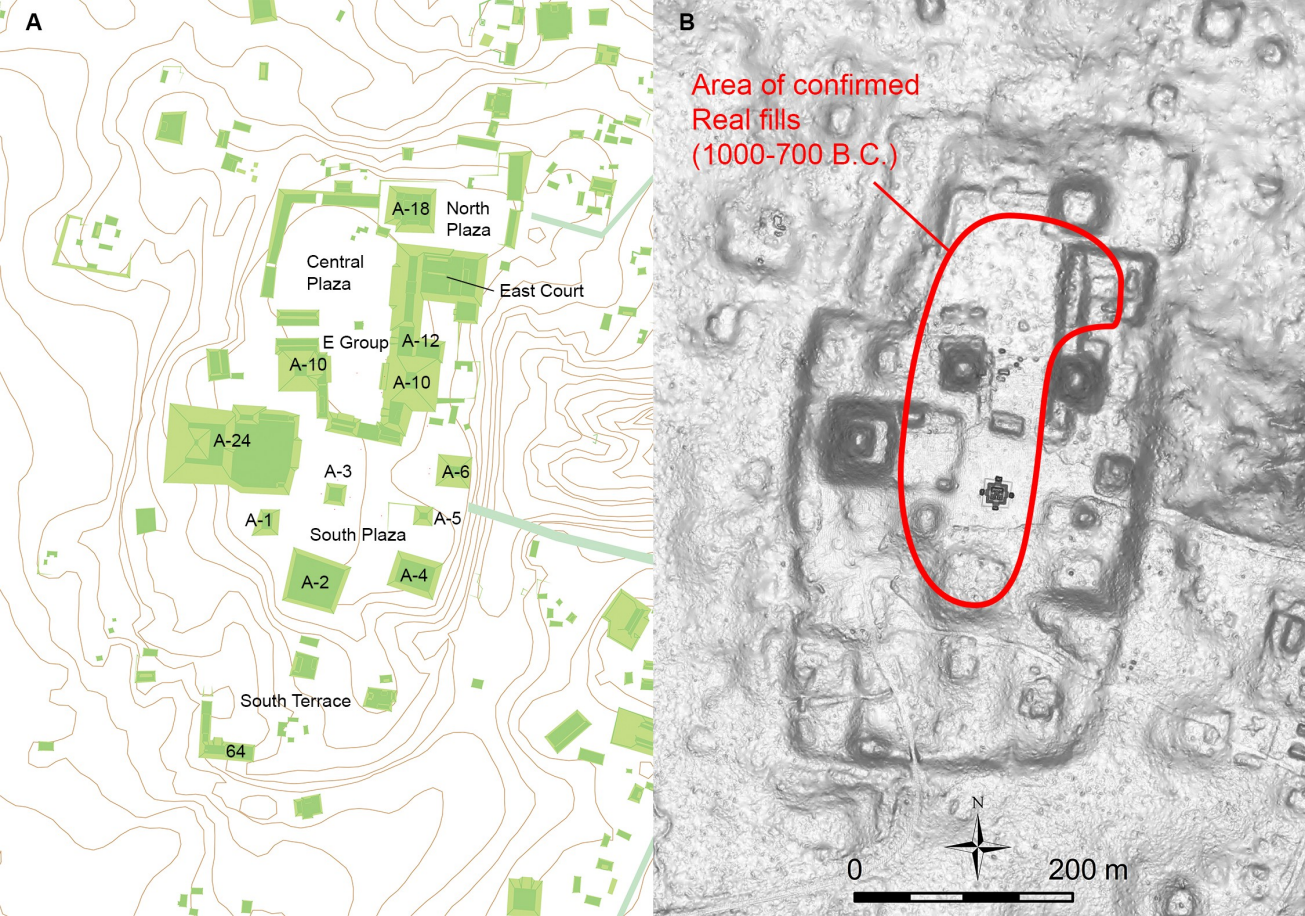
Group A plateau of Ceibal. A. Map produced by the HP (1m contour lines). Redrawn from [5]. B. LiDAR-derived DEM. The area of confirmed Real-phase fills is indicated.

An important benefit of LiDAR is that its bird’s eye views without vegetation allow us to identify large-scale landscape modifications that are difficult to see from the ground level. HP researchers produced an excellent map of Ceibal through a pedestrian survey, recording many structures accurately. This map showed the central part of the site called Group A as a raised terrain in an amorphous shape (Fig 3). HP and CPAP archaeologists knew that parts of Group A were artificially modified, but dense vegetation prevented us from recognizing its overall shape accurately. LiDAR revealed that this area was an artificial plateau in a roughly rectangular shape, measuring 600 m in length (north-south) and 340 m in width (east-west) and rising 6 to 15 m above the surrounding ground surface. We use the term artificial plateau to describe this type of massive constructions to distinguish them from supporting platforms and other smaller buildings. Supporting platforms may measure 200 x 200 m or less and usually support multiple buildings on top of them. Artificial plateaus are larger than supporting platforms in horizontal dimensions, and the Ceibal plateau underlay multiple pyramids, supporting platforms, and plazas.

### Excavations

**Fig 4:**
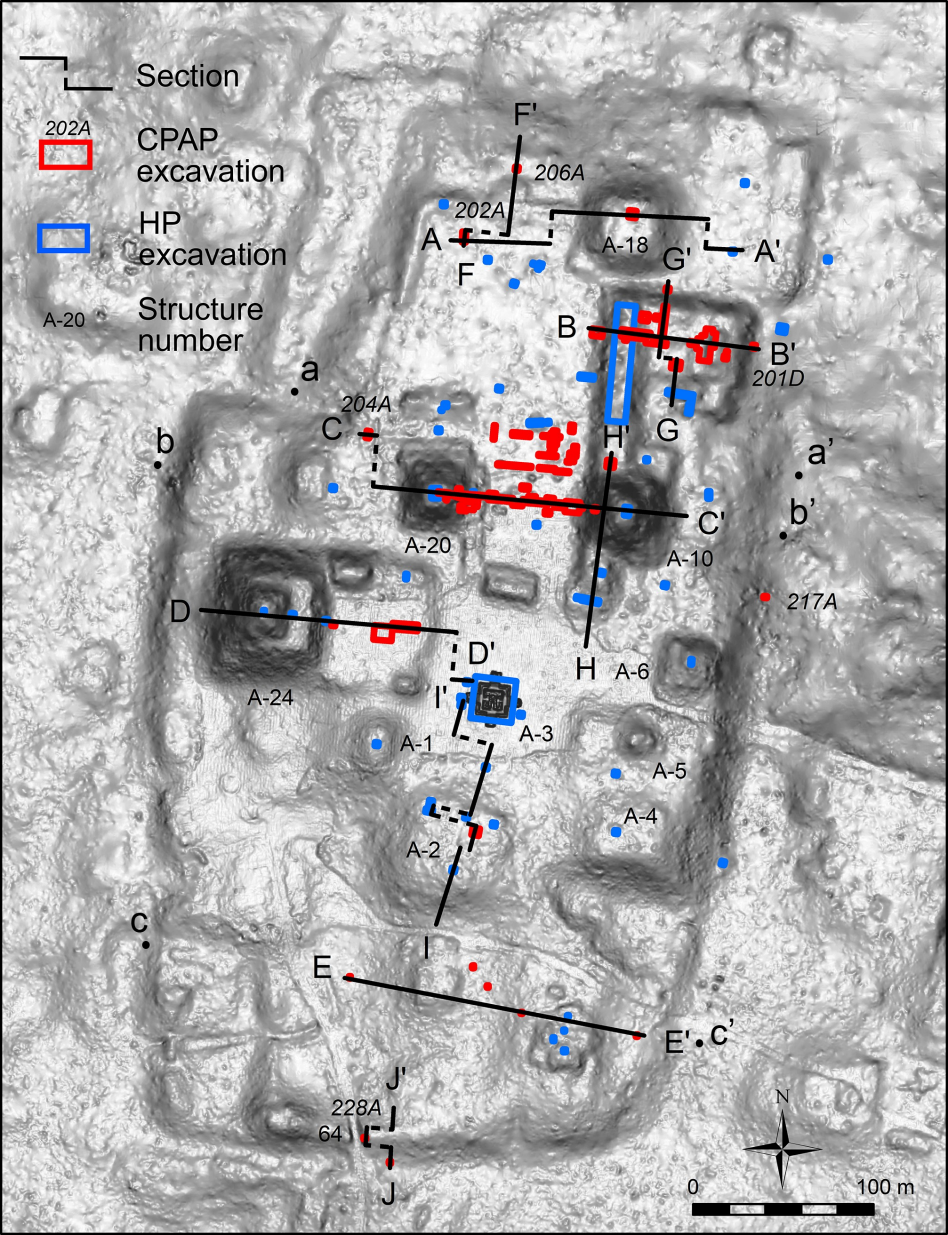
LiDAR-derived DEM of the Ceibal plateau with the locations of excavations and the lines of important sections shown in Figs 5 and 6.

Data on the construction process of the Ceibal plateau derived from numerous excavations carried out by the HP and the CPAP (Fig 4). Detailed discussions of the HP excavations are found in [7,26], and those on the CPAP in [27–33]. All excavated materials are stored at the National Museum of Archaeology and Ethnology and the Salon 3 storage facility of the Guatemalan government (7a Avenida y 6a Calle, Zona 13, Guatemala City). Researchers interested in those materials should request permission from the Institute de Antropología e Historia de Guatemala (12 Avenida 11–11, Zona 1, Guatemala City; phone +502 2208 6600; vu.demopre@gmail.com), Guatemalafor access to these facilities.

Many of the CPAP excavations penetrated through construction layers of different periods to reach bedrock. Small HP excavations in plazas were also dug to bedrock, but most HP excavations placed over structures were limited to surface layers. These results indicated that the plateau was constructed throughout the history of Ceibal, but its initial construction started at the beginning of the Middle Preclassic, and a substantial part of its fill volume was placed during the Preclassic period.

**Fig 5:**
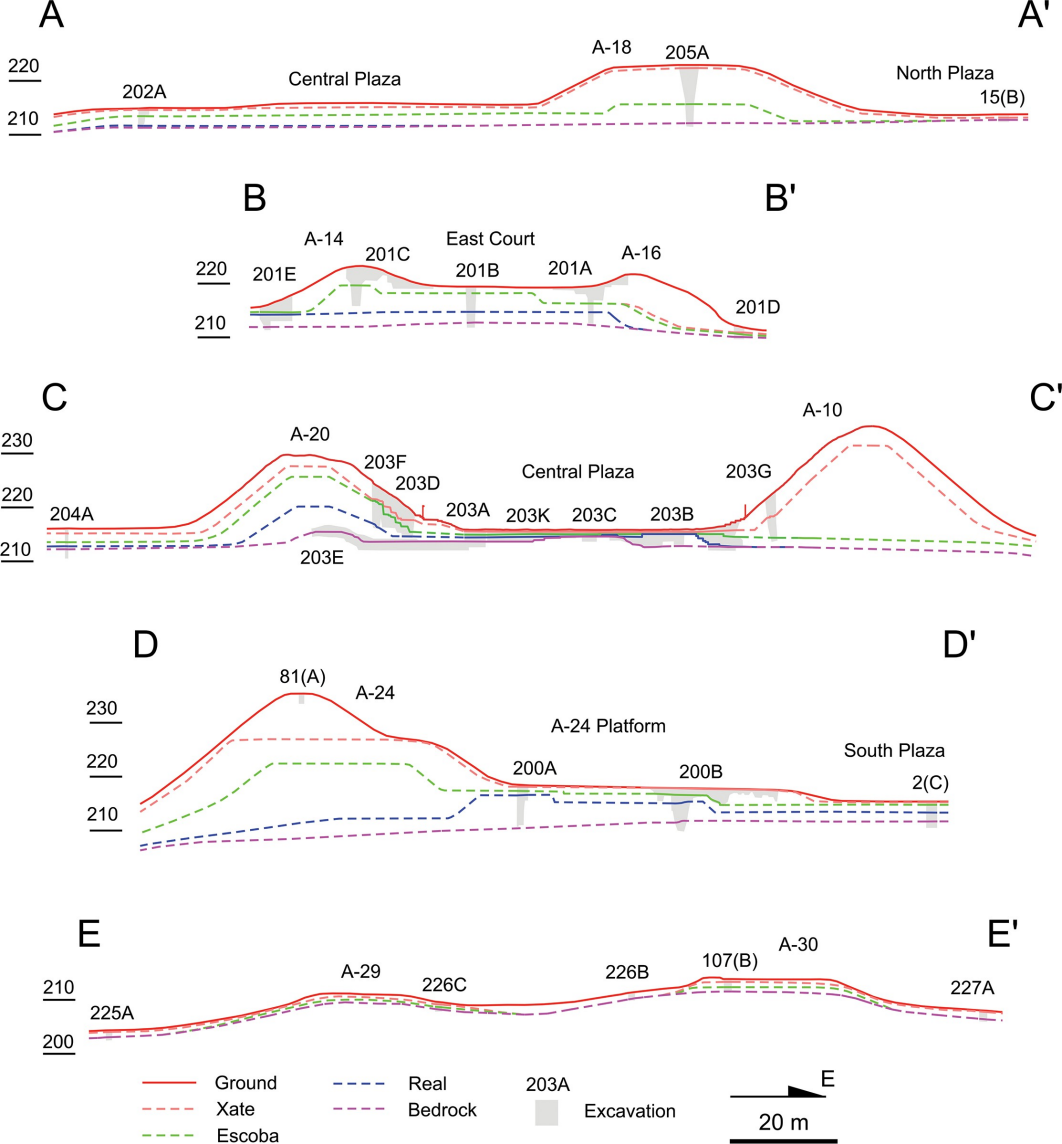
East-west lines of important sections. For the locations of these section lines, see Fig 4.

**Fig 6:**
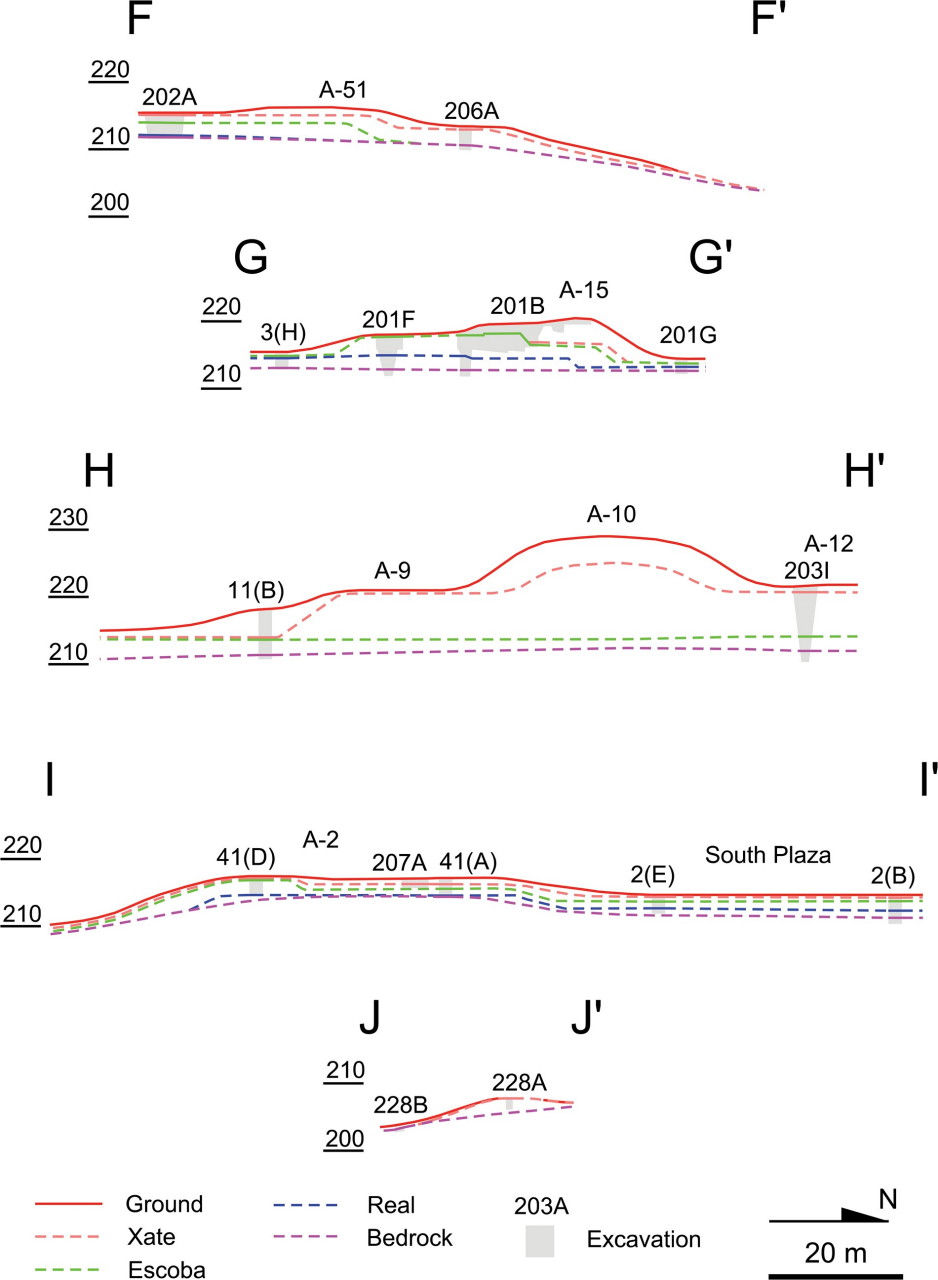
North-south lines of important sections. For the locations of these section lines, see Fig 4.

The primary focus of the CPAP excavations was the southern part of the Central Plaza, where we identified the earliest formal ceremonial complex known in the Maya lowlands. It was built in a standardized configuration called an E-Group assemblage, which consisted of a western mound and an elongated eastern platform flanking a plaza [8–10,28,34]. The earliest version of the Ceibal E Group dates to 950 B.C., and this architectural format spread throughout the Maya lowlands in later periods. At Ceibal, the first construction episode involved the scraping of surface soil and bedrock to shape the initial E-Group buildings and the plaza out of bedrock. The original stage of the plateau may also have been formed at that time through the carving of bedrock, but it is difficult to determine the date of surface scraping in the peripheral parts of the plateau. The western structure and the plaza were then raised with earthen fills at least twice during the Real 1 phase (1000–850 B.C.). Builders continued to renovate the plazas and structures by adding fills over earlier constructions in subsequent periods. The fill thicknesses of the E-Group plaza reached 1.4 to 1.6 m by the end of the Real phase and 1.8 to 2.8 m by the end of the Preclassic, whereas the Classic portions of the fills were 0.3 to 0.4 m thick (Figs 5 and 6, and Table 1). Our excavation showed that Structure A-12, the northern portion of the eastern building, measured 8.7 m in height by the end of the Preclassic, while Classic constructions added only 0.9 m. The presence of numerous caches with greenstone axes suggests that the E-Group assemblage was the primary focus of communal ritual at Ceibal during the Middle Preclassic [28,30,33].

**Table 1:** Fill thickness by period (m).

Area/structure [operation]		Real	Escoba	Cantutse-Xate	Preclassic total	Classic
		(1000–700 B.C.)	(700–350 B.C.)	(350 B.C.-A.D. 175)	(1000 B.C.-A.D. 175)	(A.D. 175–950)
Central Plaza						
	E-Group Plaza [203A, B, C, K]	1.4–1.6	0.4–0.5	0.3–0.6	1.8–2.8	0.3–0.4
	Northern part [202A]	0.4	1.8	0.9	3.1	0.4
	Western part [204A]	0.6	0.7	1.6	2.9	1.0
	Northern edge [206A]	0	0	2.4	2.4	0.5
	Northeastern edge [201D]	0	0.6	0.2	0.8	1.2
	Eastern edge [217A]	0	0	1.6	1.6	1.9
South Plaza [2(B), (C), (D)]		1.0–1.8	1.4–1.8	0.6	3.2–3.6	0.2
North Plaza [15(A), (B)]		0	0–0.2	0.6–1.0	0.6–1.0	0.4–0.6
South Terrace [225A, 226C, 227A]		0	0.4	0.6–0.8	0.7–1.2	0.3–0.6
Platforms						
	A-24 Platform [200A, B]	3.4–6.0	0.7–1.5	0.6–1.1	6.0–7.5	0.1–0.3
	East Court [200B, F]	2.0–2.1	2.7–3.3	0–0.3	4.7–5.2	0.1–0.3
	A-2 [41(A), (D)]	0.2	0.6–2.2	0–0.8	1.6–2.4	0.8
	A-4 [48(A)]	0	0.6	1.6	2.2	0.6
	A-18 [205A]	0	3.6	6.7	10.3	0.6

In the South Plaza, test pits excavated by the HP revealed even deeper Real and Preclassic fills [7]. The magnitude of Preclassic construction was also suggested by the excavations of supporting platforms. The A-24 Platform and the East Court reached heights of 3.4–6.0 m and 2.1 m respectively by the end of the Real phase, and 6.0–7.5 m and 4.7–5.2 m by the end of the Preclassic, whereas Classic-period fills were minimal. HP excavations revealed Real layers under Structure A-2. Other platforms, including Structure A-4, and A-18, appear to have been made mostly after the late Middle Preclassic Escoba phase, but substantial parts of their masses date to the Preclassic (Figs 5 and 6). These supporting platforms, arranged along the north-south axis of the E Group, formed a standardized site plan called the Middle Formative Chiapas (MFC) pattern during the Middle Preclassic period [8,29]. Contemporaneous MFC patterns are found at the Olmec center of La Venta and Chiapas sites along the Grijalva River and on the Pacific Coast [35–38].

Substantial volumes of the pyramids in Group A were also built during the Preclassic period. Although most pyramids, except Structure A-20, were not excavated down to their earliest cores, shallow excavations were sufficient to uncover Preclassic constructions in Structures A-1, A-5, and A-6. The only exception to this pattern is Structure A-3, a small pyramid in the South Plaza, which was built entirely during the Terminal Classic [7,39]. Excavation data on peripheral parts of the plateau are more limited but still indicative of Preclassic constructions. Excavations in the northern part of the Central Plaza (Op. 202A) showed evidence of surface soil scraping, floors dating to the Real-Escoba transition, and substantial Preclassic fills, covered by thin Classic layers. To the west of the E Group (Op. 204A), we also found a Real layer and substantial Preclassic fills (Figs 4–6 and Table 1). The small North Plaza, however, appears to have been added during the Escoba phase, and we did not find Real layers in excavations placed just outside of the plateau (Ops. 206A, 201D, and 217A).

**Fig 7:**
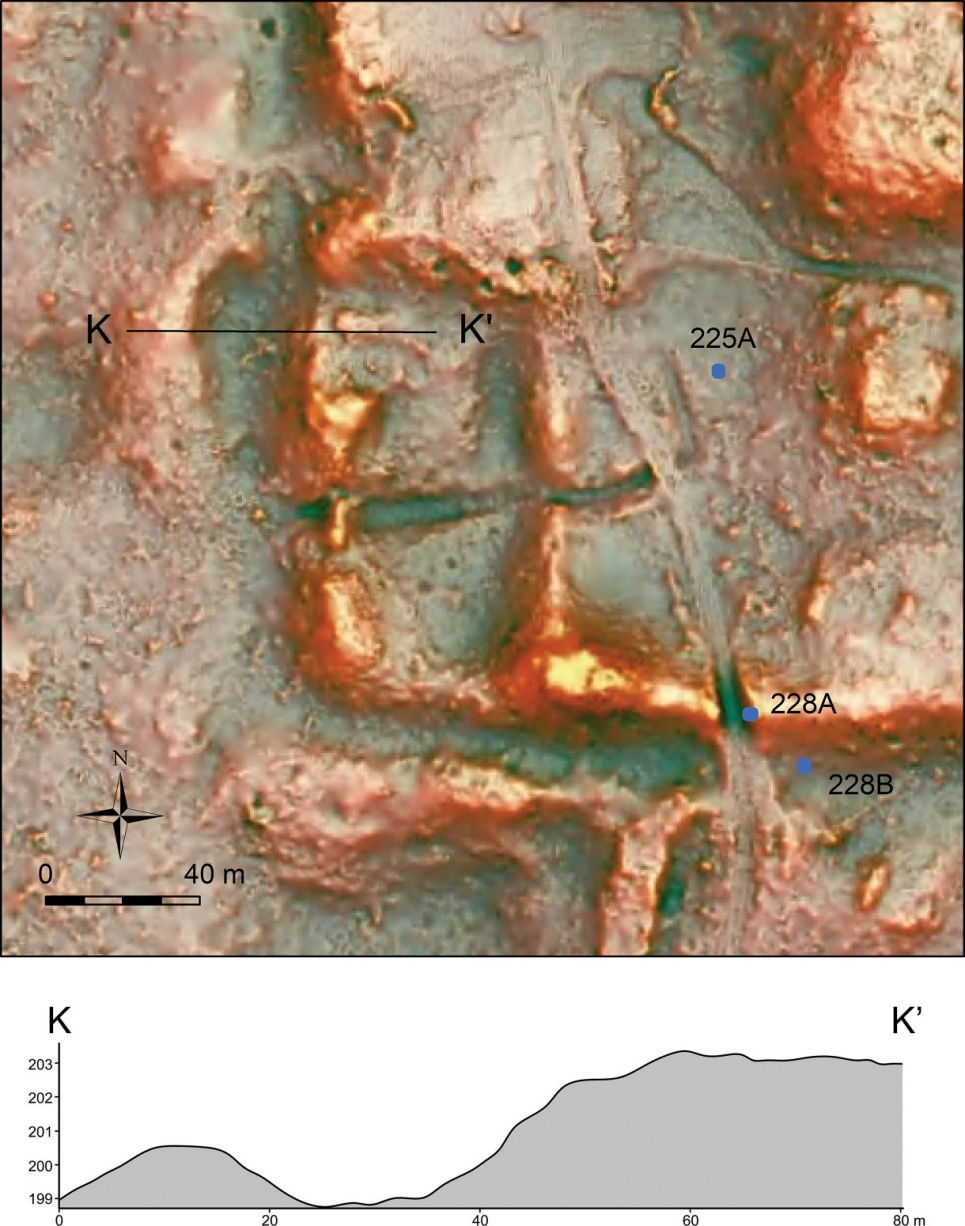
LiDAR-derived DEM in the RRIM visualization showing the southwestern part of the plateau and a section showing possible back dirt and an edge of the plateau. The vertical dimension of the section is exaggerated.

In the South Terrace of the plateau, fill thicknesses are modest, and Real fills are generally absent. It appears that the South Terrace was shaped mainly through the carving of surface soils and bedrock, particularly in its early construction stages. The LiDAR data show possible piles of back dirt along the western and southern edges of the South Terrace (Fig 7). These piles become particularly clear in the Red Relief Image Map (RRIM) visualization of the LiDAR data, which highlights subtle surface morphologies [12,40]. Builders probably dug into gentle slopes of bedrock to define the edges of the plateau with steep slopes. They seem to have dumped back dirt 15 to 30 m away from the plateau. The excavation of Operation 228B in the southern edge revealed carved bedrock under a thin layer of eroded soil (Figs 4–6). We could not determine when these edges were carved. Nonetheless, the excavation of Structure 64, a long mound along the southern edge (Op. 228A), indicates that this mound was initially constructed during the Escoba 3/Cantutse 1 phase or earlier (Figs 4–6). It is likely that the southern edge was carved and defined at least by the end of the Middle Preclassic.

**Fig 8:**
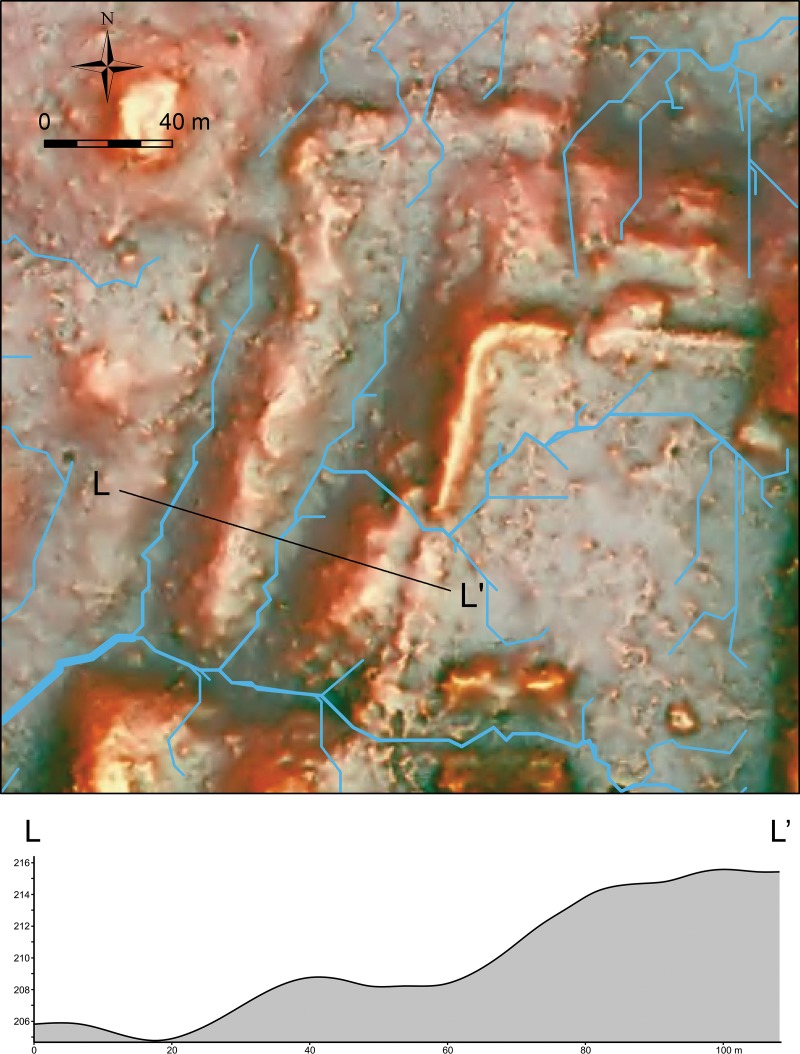
LiDAR-derived DEM in the RRIM visualization showing the northwestern part of the plateau and a section showing a berm and an edge of the plateau. The vertical dimension of the section is exaggerated. Blue lines indicate the probable flows of water reconstructed with ArcGIS’s hydrological analysis functions.

In the northwestern part of the plateau, a more regularly-shaped berm is visible roughly 30 m away from the plateau’s edge (Fig 8). We originally suspected that it was for directing surface runoff water to a depression located north of the plateau. The hydrological analysis of the DEM, however, shows that the channels along the berm led water to the north and south. Water-channeling does not seem to have been the primary function of the berm. One possible interpretation is that the berm was back dirt from the carving of bedrock in defining the plateau’s edge. Another possibility is that the Ceibal residents were preparing to expand the plateau in this area, and the berm defined the planned edge of the extended plateau. In this scenario, the builders never completed this expansion.

These data show that the initial form of the plateau was built during the early Middle Preclassic Real phase (1000–700 B.C.). The early fills in the E-Group assemblage were placed around 950 B.C., and the carving of bedrock may have taken place slightly earlier. Although we cannot define the exact shape of the Real-phase plateau, it comprised a roughly 400 x 200 m area where Real fills have been documented, consisting of the Central and South Plazas, the East Court, the A-24 Platform, and Structure A-2 (Fig 3). If areas beyond this limit, including the South Terrace, were shaped during the Real phase through the carving of bedrock, the initial stage of the plateau would have been substantially larger. By the end of the Preclassic period around A.D. 175, a substantial part of the plateau was built, and it probably resembled the current form shown in the LiDAR-based DEM.

### Estimates of fill volumes and labor investments

To evaluate the social importance of plateau construction, we estimated its fill volumes and the labor investments required for this construction. Before the present study, Inomata estimated the earth-fill volume of the Central and South Plazas in 2014 [41]. This work was done before the LiDAR survey and the detection of the plateau and thus did not include the volumes of other parts of the plateau. The present study expands Inomata’s previous work through the production of detailed 3D digital models by incorporating additional excavation data and the LiDAR data (Fig 4, S1 Text).

#### Fill volume estimates

We first calculated the total fill volume of the plateau, by subtracting the 3D digital model of bedrock estimated through excavations from the ground surface model provided by the LiDAR data. Thus, the estimate of the plateau fill volume included the fills of pyramids, supporting platforms, and other buildings located on top of it. We then separated the Preclassic and Classic portions of the fills. The Preclassic fills were further subdivided into the Real, Escoba, and Cantutse-Xate phases. Table 2 summarizes the estimated volumes of the plateau and all pyramids by period. We calculated possible lower and higher estimates for the entire fill volume and those of the Preclassic and Classic periods, but Table 2 shows what we think the most plausible estimates.

**Table 2:** Most plausible plateau volume estimates by period.

	Period		Phase duration (years)		Volume (m^3^)	Yearly average
Plateau						
	Preclassic		1175		550,587	469
		Real	300		98,827	329
		Escoba	350		203,040	580
		Cantutse-Xate	525		248,721	474
	Classic		775		161,319	208
All pyramids						
	All periods		1950		75,506	39

**Fig 9:**
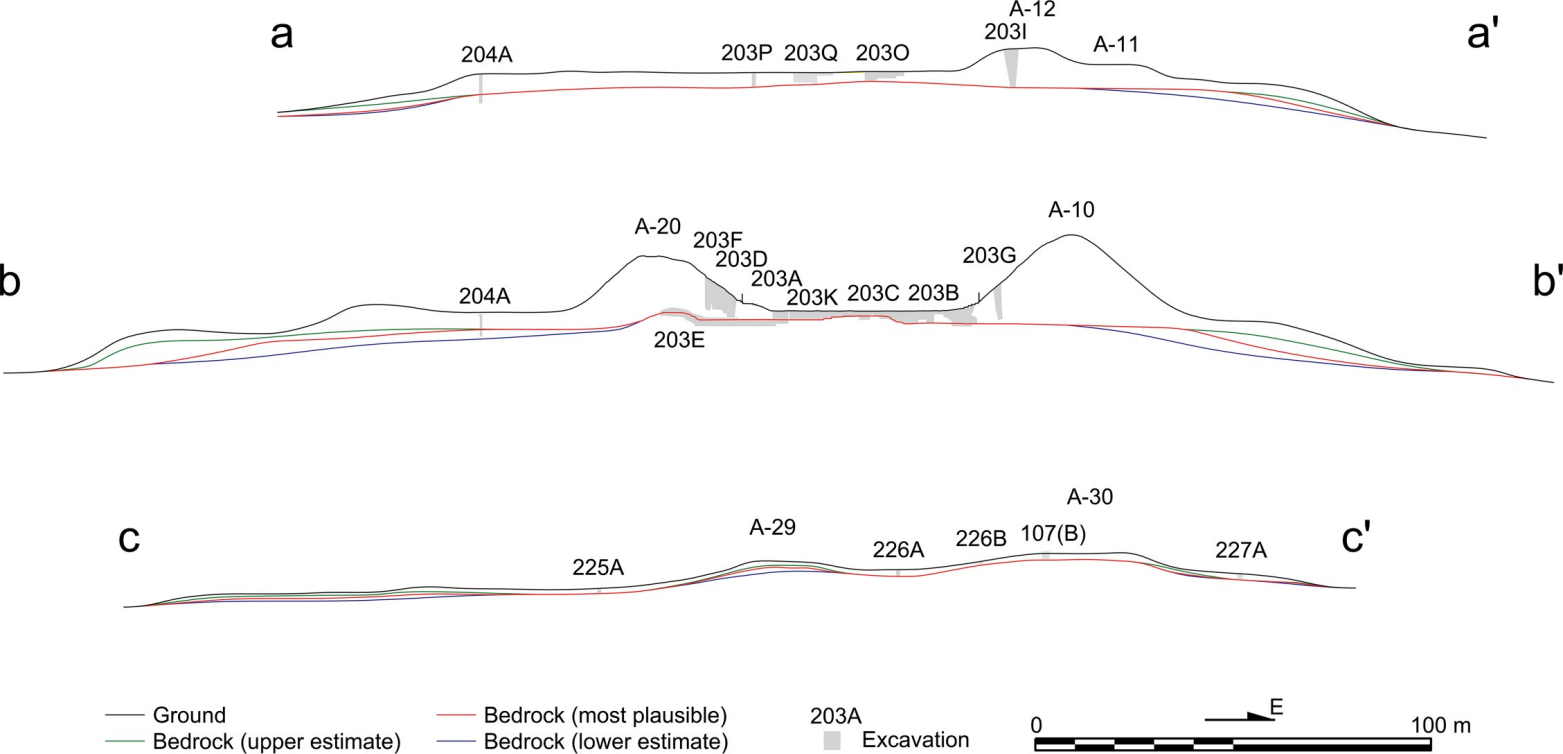
Sections showing the ground surface, excavations, and three versions of bedrock: The upper estimate (minimum plateau volume); the most plausible estimate; and the lower estimate (maximum plateau volume).

**Fig 10:**
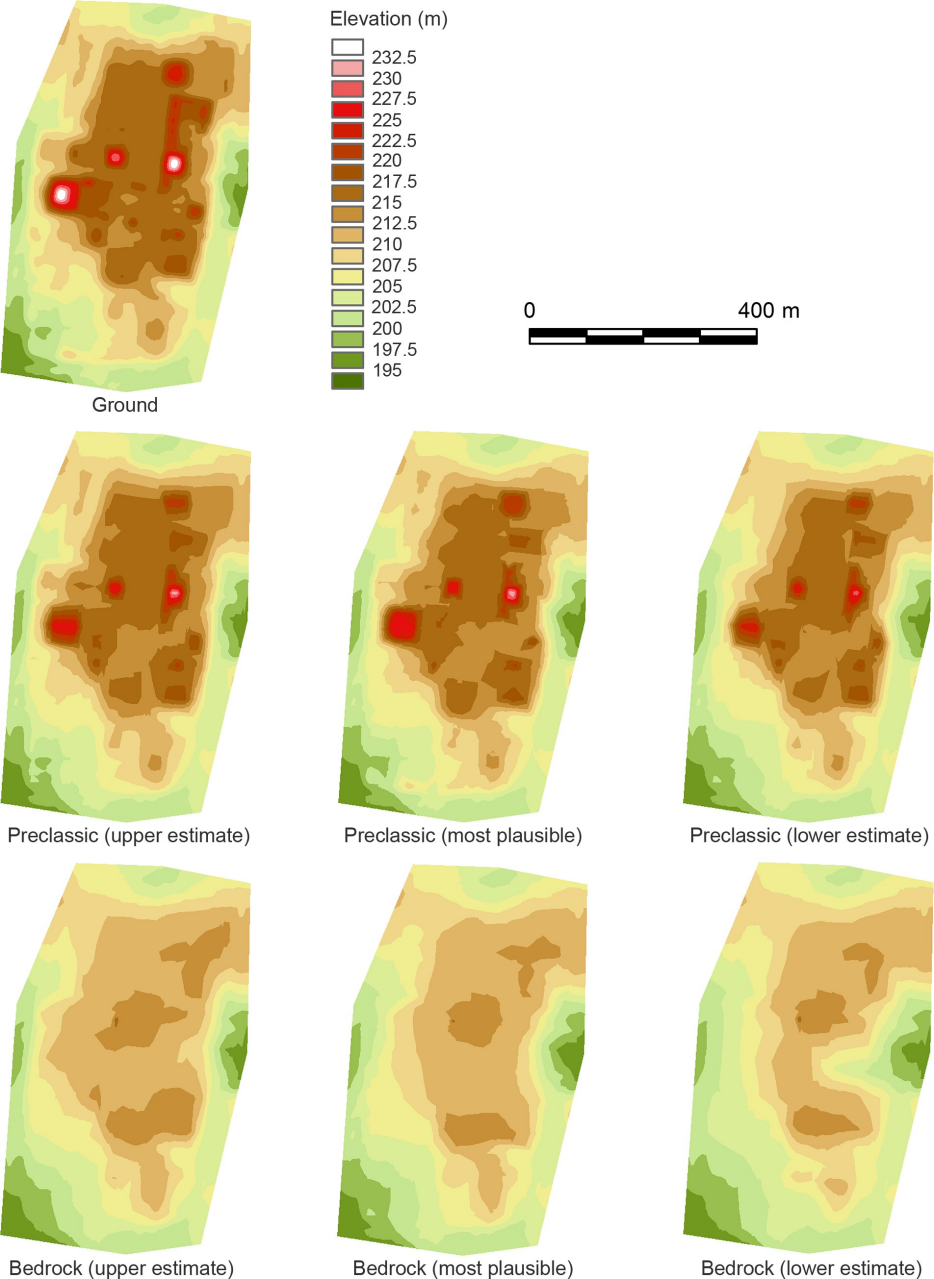
DEMs showing the upper, most plausible, and lower estimates of the Preclassic (the end of the Xate phase) and bedrock surfaces.

**Fig 11:**
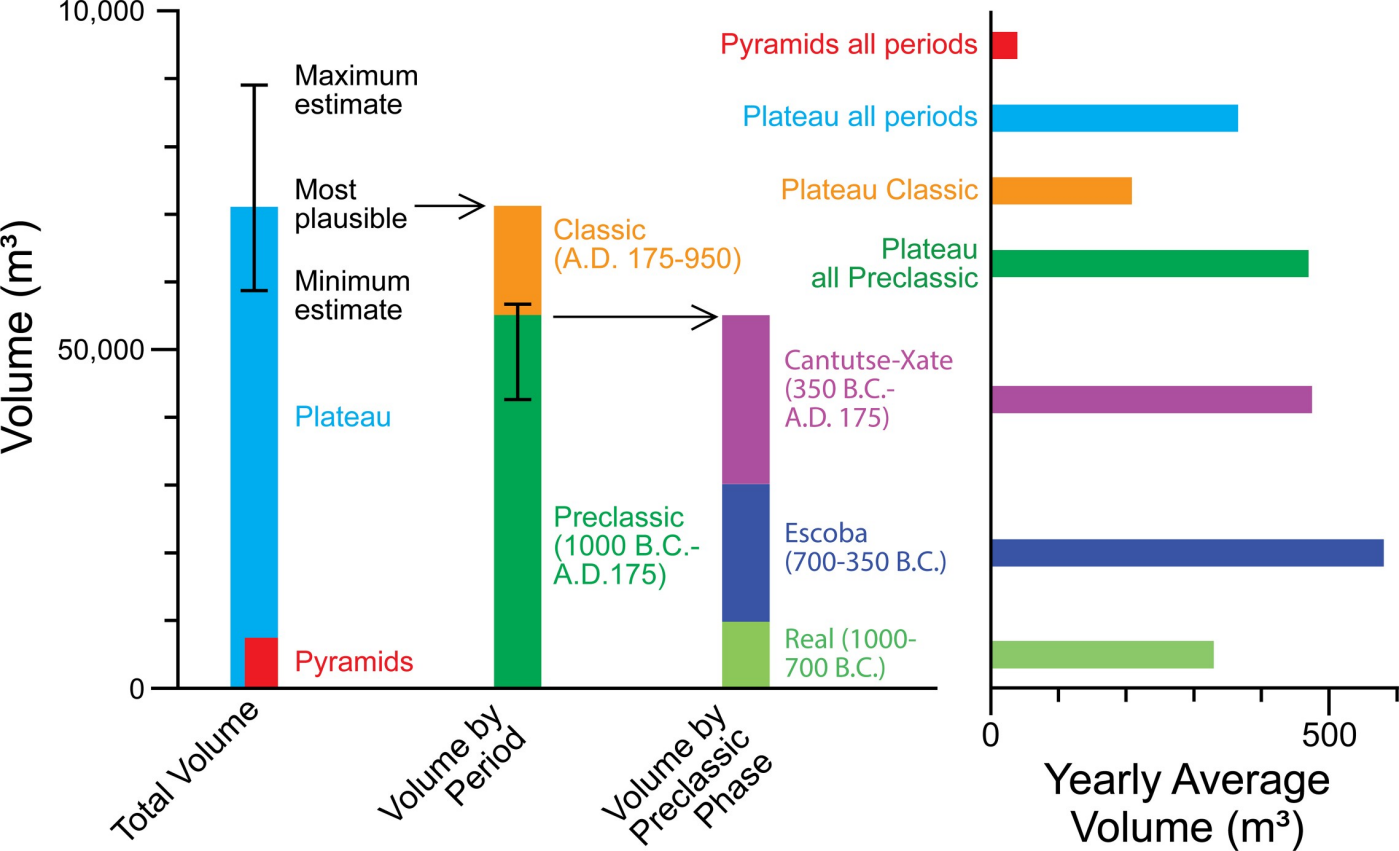
Estimates of plateau fill volumes and yearly average volumes. The volumes of pyramids are included in the plateau volumes. The volumes by period and those by Preclassic phase are based on the most plausible total volume and the most plausible Preclassic volume respectively. The yearly average volumes are calculated from the most plausible volume estimates.

In reconstructing the elevations of bedrock, we had reasonably good data for the E-Group plaza, the East Court, and the central part of the South Plaza. Data were sparser in areas outside of these complexes. To show a range of possible error in volume calculations, we first created three versions of bedrock reconstruction: 1) the minimum volume given by the upper estimated elevation of bedrock; 2) the estimate that we think the most plausible; and 3) the maximum volume given by the lower estimated elevation of bedrock (Figs 9 and 10). For the southwestern part of the plateau where excavation data are lacking, we created conservative bedrock models by placing them close to the current ground surface. Thus, it is unlikely that our estimates of the plateau volume are unreasonably inflated. We should also note that the minimum volume estimate assumes that bedrock levels were high in the peripheral parts of the plateau. This scenario would imply that builders carved and removed a substantial quantity of bedrock to shape the plateau edges. The quantities of such removed material are not included in our fill volume estimates. We then calculated the total fill volume for all pyramids located on the plateau. These data show that even in our smallest estimate, the construction volume of the plateau is substantial. The total plateau fill is 7.8 to 11.8 times larger than the combined volume of the pyramids (Fig 11, Tables A and B in S1 Text).

To calculate the Preclassic and Classic portions of the plateau fill volume, we created three versions of the Preclassic fill model based on the most plausible model of bedrock (Fig 10). In the 2014 study, Inomata assumed that construction activity during the Junco phase was negligible, but subsequent investigations identified some buildings dating to this period. In the present study, we chose to calculate the combined volume for the entire Classic period, including the Junco, Tepejilote, and Bayal phases. These estimates suggest that 59.7% to 79.3% of the total plateau volume was constructed during the Preclassic period. We should caution that if we make such estimates for the minimum and maximum bedrock models, the error range for the Preclassic volume will be larger. Nonetheless, if we add the quantity of soil and bedrock that were removed, the total amount of moved materials during the Preclassic period would have been even larger. Thus, these estimates provide clear evidence that significant construction activity took place during the Preclassic period.

**Fig 12:**
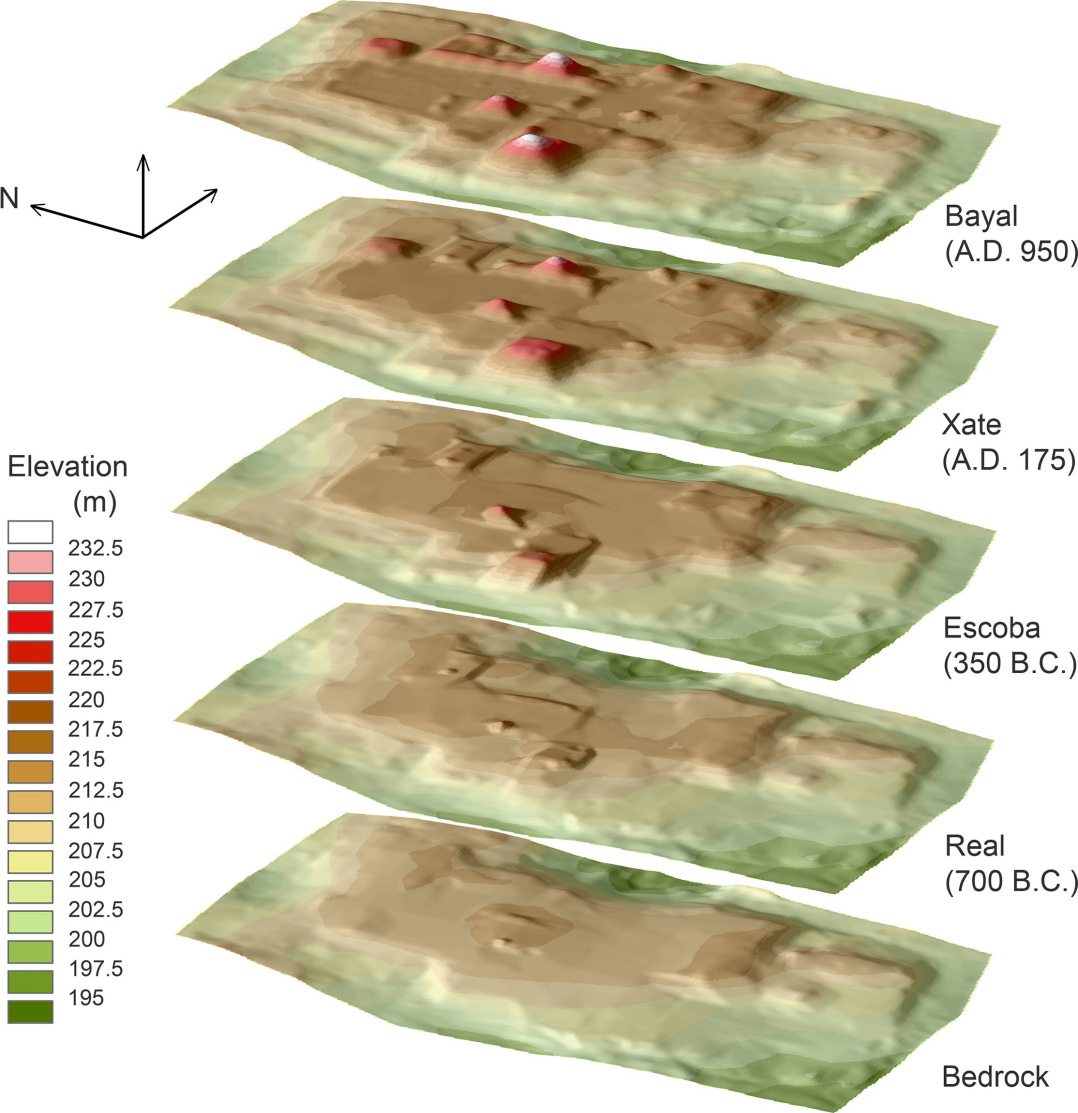
Three-dimensional digital reconstructions of the Ceibal plateau through time.

We then subdivided the second (most plausible) Preclassic model into shorter ceramic phases (Fig 12, S1 Text). We combined the Cantutse and Xate phases for volume calculations because the differentiation of ceramics from these phases was sometimes difficult. These estimates suggest that a considerable portion of the plateau was constructed during the Real and Escoba phases of the Middle Preclassic period. By 800 B.C., the A-24 Platform alone represented a monumental scale, reaching a height of 6 m and an east-west dimension of more than 34 m [8]. The mass of the entire plateau during the Real phase was substantially larger, possibly surpassing the combined volume of pyramids of all periods. Again, we need to consider ranges of error involved in these calculations, but a significant portion of the Real and Escoba fills in our models is concentrated in the central part of the plateau where we have abundant excavation data. It is unlikely that the true Middle Preclassic volume was much lower than our estimates.

#### Labor investment estimates

Based on the estimates of fill volumes, we then calculated possible labor investments required for the plateau construction. We first calculated total labor investments in person-days (Table 3). Through a comparison with estimated populations at Ceibal (Table 4), we then assessed how long these constructions took (Table 5). It should be emphasized that the ranges of error for these estimates are even larger than those for the construction volumes. Although we provide low and high estimates for each category of calculation, the true figures may be substantially smaller or larger than our estimates. These estimates should be understood as heuristic exercises, which are intended to help us explore the social implications of construction activity.

**Table 3:** Estimated labor investments in the plateau construction.

	Period	Real		Escoba		Cantutse-Xate		Classic	
		Low estimate	High estimate	Low estimate	High estimate	Low estimate	High estimate	Low estimate	High estimate
Labor (person-day)									
	Procurement	38,010	89,843	78,092	184,582	95,662	226,110		62,046	146,654
	Transport	257,625	312,273	529,291	641,564	648,373	785,907	420,531	509,735
	Filling	0	20,589	0	42,300	0	51,817	0	33,608
	Total	295,636	422,705	607,383	868,446	744,035	1,063,834	482,577	689,996
Adjustment for masonry (%)		0	2	0	5	0	8	0	10
Adjusted labor (person-day)		295,636	431,159	607,383	911,869	744,035	1,148,940	482,577	758,996

**Table 4:** Population estimates by area and period.

	Period	Real	Escoba	Cantutse-Xate	Tepejilote-Bayal
Tourtellot					
	Sampled area (15.25 km^2^)	74	508	9,618	7,577
	Ceibal center (5.41 km^2^)	47	325	6,156	4,016
	Ceibal horst (133.10 km^2^)^a^	167	1,148	21,737	24,853
Inomata et al.					
	Ceibal center (5.41 km^2^)			2,648	6,514
	Ceibal horst (133.10 km^2^)^a^			9,368	40,180

^a^The population estimates for the Ceibal horst include those for the Ceibal center.

**Table 5:** Estimates of days required for the plateau construction.

	Period	Real		Escoba		Cantutse-Xate		Tepejilote-Bayal	
		Low estimate	High estimate	Low estimate	High estimate	Low estimate	High estimate	Low estimate	High estimate
With the Ceibal center population									
	Total	15,641	45,622	4,681	14,055	303	2,169	185	941
	20 year cycle	1,043	3,041	267	803	12	83	11	54
With the Ceibal horst population									
	Total	4,418	12,888	1,322	3,970	86	613	30	153
	20 year cycle	295	859	76	227	3	23	2	9

Table 3 shows the low and high estimates for labor investments by listing costs involved in the procurement of earth used in fills, their transport, and fill constructions. The formulae for these calculations derived from earlier experimental studies by various scholars [42–46] (see S1 Text for a detailed explanation). The estimates were made based on the most plausible construction volumes for individual periods listed in Table 2. We first made these calculations, assuming that the entire plateau fill consisted of earth. Subsequently, we applied adjustments to the high estimates to account for the higher construction costs of masonry, which made up small portions of the plateau and the buildings standing on it. Our labor investment estimates were based only on the fill volume figures, and the scraping of surface soil and bedrock was not included. If we consider the costs of scraping, the total labor investments should be larger, particularly for the Real phase.

Population estimates were made previously by Tourtellot [26,47] and Inomata et al. [13] (Table 4). Tourtellot’s estimates were based on a ground survey and excavation results, whereas Inomata el al. mainly used LiDAR data. There are substantial debates about the methods of population estimates [48], and different assumptions can lead to divergent results even for the Classic period, for which we have relatively reliable settlement data. Inomata et al. calculated low and high estimates for this period, and here we use their high estimate to explore the shortest possible construction time. Estimates for the Preclassic period are even more problematic, and there is a substantial difference between the figures presented by Tourtellot and by Inomata et al. Tourtellot also suggested the possibility of a lower estimate through a time-weighted adjustment, which resulted in a Cantutse-Xate population figure similar to Inomata et al.’s [26]. Inomata et al. did not attempt to estimate populations for the Real and Escoba phases. As discussed below, Tourtellot likely underestimated these Middle Preclassic populations.

Through the analysis of LiDAR data, Inomata et al. defined zones of settlement distributions around Ceibal [13]. The structure density is high in the 5.41 km^2^ area around Group A demarcated by escarpments and poorly-drained terrains. Inomata et al. called this area the Ceibal center and assumed it to be the core of the Ceibal community. The terrain of 133.10 km^2^ surrounded by the Pasión River and wetlands is called the Ceibal horst. The residents of this area outside of the Ceibal center may have been loosely affiliated with Ceibal or may have been under its direct political control during different periods. Tourtellot estimated populations for his sampled area of 15.25 km^2^, which we converted to the figures for the Ceibal center and the Ceibal horst, by applying population ratios assessed from LiDAR data. For the Preclassic period, when the population was markedly concentrated in the Ceibal center, we estimated that the population ratio of the Ceibal center to Tourtellot’s sample area was 0.64 and the ratio of the Ceibal horst 2.26. For the Classic period, we applied figures of 0.53 and 3.28 respectively.

By dividing the total labor investments in person-days by population estimates, we obtained estimated numbers of days required for the construction of the Ceibal plateau (Table 5). We calculated two sets of estimates, one with the assumption that only the populations of the Ceibal center participated in construction and the other with the assumption that the entire populations of the Ceibal horst joined. For each set, we calculated low and high estimates. The low estimates are based on the low person-day estimates and the higher population estimates between Tourtellot’s and Inomata et al.’s, as well as the assumption that two persons from an average household of five contributed to construction. The high estimates were obtained with the high person-day estimates, the lower population estimates, and the participation rate of one person out of five. For the Classic period, we assumed that all construction volume of this period was placed during the Tepejilote-Bayal phase.

We also calculated possible construction times in individual major construction episodes. Although small-scale building activity probably took place each year or every few years, larger-scale construction events may have taken place in longer cycles. Our excavations in the central part of the plateau revealed seven floors dating to the Real phase, five dating to the Escoba phase, and five dating to the Cantutse-Xate phase, hinting at such long cycles. We calculated the days required for each of the major construction episodes, assuming that such events took place every 20 years, which corresponds to the primary ceremonial cycle of the Classic period.

Although these estimates contain large ranges of error, the results suggest that Tourtellot’s estimates for the Real populations are probably too low. It is unlikely that builders engaged in construction activity more than three or four months a year. We should reiterate that the true labor investment for this period may have been substantially larger than our estimates because our calculations did not include the labor for scraping of surface soil and bedrock. The population during the Real phase was probably substantially larger, and the Escoba population estimates may also need to be adjusted upward.

The wide discrepancies in the population estimates for the Cantutse-Xate phase make the evaluation of this period difficult. However, it is reasonable to think that the construction of ceremonial buildings during this period was a significant social endeavor. During this period, many temple pyramids outside Group A were built, adding to labor demands. In comparison, the expansion of the plateau during the Classic period required a much smaller portion of social resources.

### Political centralization

We have discussed political processes at Ceibal in our previous publications [9,13,28,29,34,49,50], and here we summarize main points. Some archaeologists use the scales of monumental buildings and population sizes as indicators of political centralization and social hierarchy [51–53]. The purpose of this paper, however, is to examine the possible emergence of monumental constructions before the development of powerful elites, and thus we need to evaluate the degrees of political centralization and social inequality through other sets of data. We focus on large and elaborate residential complexes that may have been used by elites, rich burials, and material symbols of political authority.

In this brief review, we emphasize the following three points. First, during the early Middle Preclassic Real phase, when the initial version of the Ceibal plateau was built, emergent elites may have existed, but their power was probably limited. Second, during the late Middle Preclassic Escoba phase, Ceibal probably did not have rulers comparable to those of centers on the Gulf Coast and in the Grijalva River region. Third, during the Late-Terminal Preclassic Cantutse-Xate phase, rich tombs and symbols of authority used by later Maya rulers began to emerge in the central lowlands, but recognizable signs of marked inequality and political centralization are absent at Ceibal. These observations indicate that a substantial part of the Ceibal plateau was built before the development of highly centralized power.

Before 1000 B.C., the inhabitants of the Maya lowlands led mobile lifeways without the use of ceramics, combining heavy reliance on wild resources with the cultivation of maize and other crops [54]. Archaeologically-recognizable features from this period are scarce, not to mention any material remains indicating marked social inequality. Around 1000 B.C., the occupants of the Maya lowlands began to adopt a more sedentary way of life, along with greater reliance on maize agriculture and ceramic use. Most scholars agree that during the subsequent Middle Preclassic period, initial processes of social differentiation were underway, but the degree of hereditary inequality was still small [55–57]. Ceibal may have been a community with more internal inequality than other Maya settlements, but the power of community leaders was most likely constrained. The presence of numerous caches with greenstone axes at Ceibal implies that some individuals played a leading role in obtaining these precious objects and organizing public ceremonies. Burial CB136, placed behind the eastern building (Structure Sib’) of the E-Group assemblage during the Real 3 phase, may have been an interment of such an individual, but it was not a particularly rich burial. It contained five ceramic vessels of common types, but no ornaments or exotic goods. A vexing problem is the nature of the A-24 Platform. It is not clear whether this large platform, built during the Real 1 phase and supporting multiple structures, was a residential complex of elite households or whether it was a communal compound used for collective events, such as meetings and feastings. Platform K’at, constructed during the Real 3 phase and later covered by the East Court, was the first case of a more clearly recognizable residential complex occupied by emergent elites [8,29].

During the Real phase, some groups may have adopted a sedentary lifestyle, but a significant portion of the Ceibal population appears to have lived in pole-in-ground structures without basal platforms, possibly changing their residences seasonally or every few years. Various anthropological studies suggest that it is difficult to impose coercive power on mobile or semi-mobile groups, who can vote with their feet [58–61]. It is unlikely that the emergent elites of Ceibal had strong power over its population. During the subsequent Escoba phase, a larger number of more durable residences with basal platforms began to appear in the immediate vicinity of Group A. The practice of rebuilding residences in the same locations over generations, which characterized Classic-period Maya society, was probably gaining popularity during this period. Burial CB104, dating toward the end of the Escoba phase, contained obsidian blades and a core, a possible shell ink pot, and shell ornaments. The buried individual was probably of high status. Nonetheless, we have not found elaborate tombs, carved monuments (coarsely-shaped uncarved monuments are present), or other signs of highly centralized power at Middle Preclassic Ceibal.

The absence of such features at Ceibal contrasts with the Gulf Coast center of La Venta and the Grijalva center of Chiapa de Corzo. At La Venta, elites expressed their power through elaborate stone sculptures and possible tombs [62,63]. At Chiapa de Corzo, elaborate Middle Preclassic tombs were found in the western structure of the E Group, as well as in other buildings [37,64]. Ceibal and Chiapa de Corzo shared remarkable similarities in building arrangements and the placements of caches [30,33]. If powerful individuals that may be called rulers existed at Middle Preclassic Ceibal, we would expect to find their tombs in locations comparable to those at Chiapa de Corzo. Our tunnel excavation through the western E-Group structure, however, did not reveal any interments. The level of political centralization at Ceibal and other lowland Maya communities was probably significantly lower than those on the southern Gulf Coast and in the Chiapas Grijalva region [38].

At the end of the Middle Preclassic period, La Venta collapsed, and some Grijalva centers experienced depopulation or political disruptions [37]. As a pivotal place of communication between the Maya lowlands and Chiapas during the Middle Preclassic period, Ceibal may have suffered impacts of these changes in the surrounding regions more strongly than other lowland settlements. The possible elite residential complexes of the East Court and the A-24 Platform were transformed into open, flat areas at the beginning of the Late Preclassic Cantutse phase (350–75 B.C.). It is not clear where community leaders of Ceibal lived during the Cantutse phase. Although the population of Ceibal grew and residential groups spread to a wider area [12,26], we have not found rich burials from this period.

The main stage of political centralization in the Maya area probably shifted to the central lowlands. The construction of large pyramids at El Mirador and other centers, as well as the San Bartolo murals with glyphs and elaborate images, suggest that early forms of the practices and symbols tied to rulership may have been in development [65–67]. The specific nature of political authority during this period, however, is still debated. It is not until the Terminal Preclassic period (75 B.C.-A.D. 250) that we recognize well-established rulers, along with unequivocal royal tombs, at Tikal and other centers [68]. The process of political centralization at Ceibal was probably slower. During the Terminal Preclassic Xate phase, Group D was constructed as a new focus of elite activity at Ceibal, but the scales of its pyramidal constructions were smaller than those at central lowland communities [7,69]. The first historically-known Ceibal ruler, who is retrospectively mentioned in a text, dates to A.D. 415 [70].

## Discussion

The earlier tradition of plateau construction is found outside the Maya lowlands, at the Olmec center of San Lorenzo, which reached its heyday between 1400 and 1150 B.C. [71–73]. The initial plateau construction at Ceibal around 950 B.C. probably represents one of the first adoptions of this tradition in the Maya lowlands. Roughly two centuries later, an E Group and a plateau were constructed at Cival. In contrast to the gradual buildup of the Ceibal plateau, a substantial amount of the hilltop leveling at Cival appears to have been accomplished in a short time span. Estrada-Belli estimates that a Middle Preclassic volume of 1,304,000 m^3^ and a Late Preclassic volume of 556,000 m^3^ were placed [4]. Early platforms at Komchen, Yucatan, may be roughly contemporaneous with Cival [74]. As the Ceibal plateau continued to grow throughout the latter half of the Middle Preclassic and the Late Preclassic, large platforms were constructed at other Maya settlements, including Nakbe, El Mirador, and Yaxnohcah in the central lowlands, and Yaxuna, Xocnaceh, and Izamal in the northern lowlands [1,75–77]. Given the difficulty of recognizing artificial plateaus covered by rainforest, similar constructions may remain undetected at other Maya sites.

Outside of the Maya lowlands, excavations at the Gulf Coast center of La Venta revealed artificial fills measuring 1.5 to 2 m in thickness in the Ceremonial Court of Complex A [58,78]. At the MFC site of San Isidro in the Grijalva River region, Early and Middle Preclassic fills of the E-Group plaza measured roughly 2 m in thickness [79]. Although it is not clear whether La Venta and San Isidro had well-defined plateaus comparable to that of Ceibal, the substantial fill accumulations in plazas or other open areas suggest that their residents shared similar practices of artificially creating extensive ceremonial spaces.

Early plateau constructions in the Maya lowlands occurred during the transition from mobile lifeways and mixed subsistence strategies without the use of ceramics to a more sedentary way of life with greater reliance on maize agriculture and ceramic use. HP researchers assumed that the Real people formed a small community nucleated around Group A and that there was no settlement hierarchy in the region [26,39]. Our study suggests that the Real population estimate based on this assumption is too low. Although our estimate of the plateau construction during the Real phase contains a wide range of error, we can reasonably state that the sacle of its construction necessitated a larger population. It was likely that there were semi-mobile groups in areas outside the Ceibal center, even though their archaeological traces are extremely difficult to detect. These peripheral groups probably participated in construction events and rituals that were periodically held at Ceibal. In this sense, Ceibal was a primary ceremonial center within a broader network of settlements, which attracted a significant number of people from surrounding areas on certain occations.

Emergent elites may have been present at Ceibal during the Real phase and may have played a leading role in organizing this unprecedented construction project. Social inequality at Ceibal, however, was still limited, and it is unlikely that community leaders possessed coercive power over groups that retained some degree of residential mobility. The initial mechanism for the construction of the Ceibal plateau was likely not coercion by elites but willing participation by community members, possibly motivated by the excitement of creating a new building on an unprecedented scale and of taking part in public ceremonies held there.

Plateau constructions may be characterized as horizontal monumentality, which contrasts with the verticality of pyramids. Horizontal building forms may have been preferred by early communities in the Maya lowlands with unpronounced social differentiation. Emphasis on horizontal monumentality is also found in other non-centralized societies in the world, such as prehistoric bison hunters of the North American Plains [80]. These observations are not meant to equate certain built forms with levels of political centralization. San Lorenzo with a large plateau, for example, had established rulers. In opposite examples, some archaic societies without pronounced social inequality, such as those in the American Southeast, built tall mounds reminiscent of pyramids (see below) [81]. With these cautions in mind, we suggest that plateaus with extensive space and open access are more conducive to inclusive interaction than pyramids, which tend to limit access to their summits to the privileged few.

The earliest pyramids in southern Mesoamerica are found outside the Maya lowlands on the Pacific Coast of Chiapas and Guatemala, where the level of political centralization was more pronounced, toward the end of the Early Preclassic and at the beginning of the Middle Preclassic [82–84]. The Middle Preclassic residents of Ceibal and Cival also built pyramids, but their volumes were substantially smaller than those of the plateaus. As shown by the example of Ceibal, the tradition of plateau construction continued in the Maya lowlands during later periods. For example, the basal plateau supporting the Danta complex of El Mirador, dating to the Late Preclassic, has dimensions similar to the Ceibal plateau, measuring 600 x 310 m horizontally and 10 to 11 m in height [65]. Nonetheless, pyramids as foci of collective construction activity gradually overshadowed plateaus after the late Middle Preclassic period. In particular, enormous pyramidal buildings at Nakbe, El Mirador, and other sites in the central Maya lowlands may have been correlated with increasing political centralization in that region [1,2,85].

This trend led to the construction of steep pyramids as dominant built forms during the Classic period, which served as symbols of now well-established dynastic rule and as places of elite burials. Pyramids at Ceibal, however, were substantially smaller than the Preclassic portions of the plateau in terms of construction volume. The Preclassic constructions of the Ceibal plateau are impressive even when they are compared to better-known examples of Classic-period pyramids. Webster and Kirker, for example, estimated the construction volume of Tikal Temple 1 and Copan Temple 26 at 18,260 and 31,905 m^3^ respectively, and their labor investments at 90,000 and 124,000 person-days [45]. Even if the true construction costs of the Tikal and Copan pyramids were significantly higher than the estimates made by Webster and Kirker, we can say that Preclassic constructions of the Ceibal plateau, even that of the Real phase, surpassed Tikal Temple 1 and Copan Temple 26 in terms of construction volume, and perhaps with regard to labor investment as well. Obviously, many other buildings were constructed at Tikal and Copan during the Classic period. Still, when we consider that the populations of Tikal and Copan during the Classic period were substantially larger than Preclassic-period Ceibal, the social importance of the Ceibal plateau during the Preclassic period is evident.

The construction of the Ceibal plateau may be comparable to early monumental constructions in other parts of the world, which emerged before or during the transition to agriculture or sedentism. Examples include: Göbekli Tepe and related Neolithic sites in the Near East; Watson Brake, Poverty Point, and other mound complexes in the Archaic lower Mississippi area; Sechín Bajo, Caral, and other monumental constructions in the Andes; and large shell mounds in the southeastern US and Brazil [81,86–91]. Although archaeologists traditionally associated monumental architecture with societies with pronounced political centralization and social hierarchy [51], these examples force us to reconsider this assumption [92,93].

We should note that there are certain differences between the Ceibal plateau and these early monumental constructions from other parts of the world. First, Ceibal dates later than most of these cases. Whereas Göbekli Tepe, Watson Brake, and other examples emerged before or during the period of initial plant and animal domestications in the regions, Ceibal postdates the domestication of maize, beans, squash, and other key crops in Mesoamerica by several millennia. Second, while the earliest monumental buildings in the Near East and American Southeast were constructed probably by groups without marked hereditary inequality, Ceibal during the early Middle Preclassic period possibly had emergent elites, albeit with limited power. In addition, the residents of Ceibal were most likely aware of the presence of more hierarchical groups on the southern Gulf Coast and in Chiapas.

A factor contributing to the late emergence of monumental constructions in the Maya area may be the nature of subsistence strategies related to maize productivity. For a long period after the initial domestication, maize cob size remained small, and maize was a small part of the diet in Mesoamerica [94,95]. Mesoamerican groups continued to rely heavily on wild resources and maintained a high degree of mobility in many regions long after the initial domestication of maize. The inhabitants of the Maya lowlands were particularly slow to adopt sedentary lifeways [96]. Various scholars suspect that around 1000 B.C. or slightly earlier, the productivity of maize increased, stimulating greater reliance on this crop [97–101]. The construction of the Ceibal plateau began at this monument of profound change in subsistence strategies and lifeways.

In this regard, the social condition surrounding the emergence of the Ceibal plateau may not be dissimilar to that of Göbekli Tepe. In both cases, unprecedented construction activities appear to have been motivated by subsistence changes: initial domestication in the case of Göbekli Tepe, and the intensified production of an existing crop at Ceibal. Large-scale construction events in those areas probably mediated collaboration and negotiation among diverse groups during the period of radical change in lifeways [50]. We should note that such changes are less obvious in the Andes and American Southeast. In the former, the combination of hunting, fishing, gathering, and horticulture persisted for millennia, whereas in the latter, the builders of monumental structures relied on wild resources without the benefit of agriculture. Nonetheless, the emergence of some centers in those regions may have been associated with rapid increases in population and intensified resource use [92,102].

These variabilities in social circumstances provide important clues to underlying processes related to early monumental constructions from across the world. An important factor in this regard may be seasonal ritual gatherings [92]. Mobile or semi-sedentary groups possibly gathered periodically and worked together to built structures on unprecedented scales. These early monumental constructions represented transformations of the lived landscape, which possibly mirrored the emergence of a new social order tied to the new ways of life [8,38,103]. The construction of prominent landmarks also paralleled people’s changing relationships to lands and landscapes. In the course of these construction projects, participants may have developed new organizational forms that coordinated different responsibilities among them. These construction events and large public gatherings probably instigated and framed new modes of political negations among participants regarding affairs of common interest. Inomata et al. have analyzed this process by applying the concept of the public sphere [50,104]. These events may also have encouraged participants to adopt new subsistence strategies and new lifestyles in some cases. In other words, we need to examine not only how political and economic bases enabled large-scale constructions but also how early building projects stimulated later political and economic changes.

## Conclusion

Preclassic artificial plateau constructions in the Maya lowlands have received little attention from researchers. This is partly because such extensive features covered by the rainforest are difficult to recognize and because archaeological studies strongly focused on pyramids, which became the hallmarks of Mesoamerican civilizations in later periods. Our analysis of the artificial plateau at Ceibal demonstrates that its Preclassic constructions were substantially larger than pyramids and other Classic-period buildings. The Ceibal plateau was constructed at the time of social transformation from mobile lifeways relying on mixed subsistence strategies without the use of ceramics to full sedentism with a strong commitment to maize agriculture and ceramic use. The construction volume of the Ceibal plateau and its labor investment during the early Middle Preclassic Real phase suggest that this scale of construction cannot be explained with the previously-proposed population size of this community. The Real-phase construction events likely involved groups living outside of the Ceibal center, who possibly retained certain levels of residential mobility and whose traces are difficult to detect archaeologically.

Increasing data on artificial plateaus and platforms at Ceibal and other lowland Maya sites show that monumental constructions existed in the Maya lowlands before the development of strong coercive power held by centralized authorities. Although archaeologists sometimes use the scales of buildings as proxies for political centralization [51], these examples cast doubt on this assumption. We need to explore not only how monumental constructions resulted from political centralization in certain contexts but also how large construction projects stimulated social changes. These artificial plateaus transformed the lived landscape of the community and provided settings for public gatherings and rituals. Large constructions probably reflected and promoted the emergence of a new social order tied to the new ways of life. The creation of an ordered yet inclusive space may have motivated many individuals to participate in unprecedented construction endeavors, in the absence of coercion by elites. At the same time, such collective work on a large scale likely required and promoted organizational and managerial innovations among participants, which may have set the stage for later administrative centralization.

These new data from the Maya lowlands add to the emerging understanding that monumental structures were built before or during the transitions from hunting, gathering, and fishing to agriculture or mobile lifeways to sedentism in various parts of the world. The Ceibal plateau, which was constructed during the intensification of maize cultivation rather than initial plant domestication, reflects diversity in social circumstances surrounding early monumental constructions across the world, as well as their commonality in their emergence before the development of marked political centralization.

## Supporting information

S1 Text Volume and labor calculations. (DOCX)
