# Supplementary material for: Artificial plateau construction during the Preclassic period at the Maya site of Ceibal, Guatemala
Source: PLoS One. 2019 Aug 30;14(8):e0221943. doi: 10.1371/journal.pone.0221943 (PMC6716660; doi:10.1371/journal.pone.0221943)
Supplement: S1 Text — (DOCX) [file pone.0221943.s001.docx]

S1 Text. Volume and labor calculations

Artificial plateau construction during the Preclassic period at the Maya site of Ceibal, Guatemala

Takeshi Inomata, Daniela Triadan, Flory Pinzón, and Kazuo Aoyama

## Most Plausible Plateau Volumes

We first created 3D models for different periods that we considered most plausible. They included: 1) the bedrock surface at the beginning of the Real phase (after the removal of surface materials); 2) the plateau surface at the end of the Real phase; 3) at the end of the Escoba phase; 4) at the end of the Xate phase; and 5) at the end of the Bayal phase (approximated by the LiDAR-based DEM).

The creation of 3D models involved the following steps. First, we drew sections of the plateau for each of the aforementioned periods, along 21 east-west lines and 14 north-south lines, using Bentley Microstation V8i CAD software (Fig A). This step started with the drawing of the surfaces documented in excavations (Table 1). We then drew estimated surfaces in unexcavated areas, using the 3D B-spline Curve function of Microstation. In estimating these surfaces, we used the following assumptions: pyramids usually had tall pedestal shapes with roughly square footprints; platforms commonly had flat pedestal shapes with mostly leveled summits and square or rectangular footprints; and plazas typically had leveled surfaces (Figs 5 and 6). We also added diagonal section lines and estimated contour lines for pyramids and platforms to make accurate representations of built forms.

Second, we extracted elevation points along each section and contour line, using the Extract Node Points from Curve function. Third, these points were imported to ArcGIS 10.5 with the CAD to Geodatabase function. Fourth, with the Create TIN function of ArcGIS, we generated a triangular irregular network (TIN) for each period based on the imported elevation points. Fifth, we created 1 m DEMs, using the TIN to Raster function with the linear method. To use the DEM of the current ground surface as the model for the end of the Classic period, we resampled the original 0.5 m DEM to a 1 m cell size. To reduce noise in the LiDAR data, we then smoothed the 1 m DEM, using the Focal Statistics function to obtain the averaging for a circle neighborhood of a five cell radius. Before calculating volumes, we clipped the DEMs of all periods with a common boundary set around the plateau. The resulting models are shown in Fig 12.

The calculation of earth-fill volumes started with the Classic period. We first created a raster representing the difference between the current-ground DEM and the Xate model with the Minus function of ArcGIS. The extrapolation of the Xate surface model from elevation points created some areas higher than the current-ground DEM. These areas had negative cell values in the difference raster. Using the Con function, we created a rectified Xate model, which retained the cell value of the original Xate model when the value of the difference model was 0 or positive and received the cell value of the current-ground DEM when the value of the difference raster was negative. We then created a rectified difference raster between the current-ground DEM and the rectified Xate model. This was followed by the calculation of the volume of the rectified difference raster with the Surface Volume function. The resulting value represented the earth-fill volume for the Classic period.

The same procedure was then applied to the rectified Xate model and the Escoba model, the rectified Escoba model and the Real model, and the rectified Real model and the bedrock model (Tables A and B). We also calculated the yearly average volume for each period, which represented an adjusted value for the length of the period. It was calculated as: (the total volume for each period) / (the length of the period in years).

## Maximum and minimum volume estimates

To understand the ranges of error comprised in our volume calculations, we also calculated maximum and minimum volumes of the plateau. For this purpose, we created 3D models representing the possible upper and lower estimates of bedrock surfaces, assuming that bedrock did not have significant irregularities (Figs 9 and 10). The upper estimate for bedrock gave the minimum total volume for the plateau, and the lower estimate led to the maximum volume (Fig 11 and Table A). We then created models of the possible upper and lower estimates of the Preclassic (the end of the Xate phase) surface (Fig 10). The maximum and minimum volumes for the Preclassic period were calculated based on the most plausible model of bedrock discussed above. To generate these models, we drew sections of bedrock and the Preclassic surfaces along the east-west lines only. The rest of the procedure was the same as the one used for the most plausible models.

## Removed material

Our volume calculations do not include the quantity of soil and bedrock removed by the Ceibal builders. The removal of surface soil at the beginning of the Real phase is well documented in the E-Group plaza and its surroundings. In addition, the LiDAR data on the southwestern and northwestern parts of the plateau suggest that the plateau’s edges were shaped by the removal of surface soil and the carving of bedrock (Fig 7). Nonetheless, we do not have specific data with which to determine the date of bedrock carving in these parts. Similar bedrock-carving may have occurred in the eastern part of the plateau. Bedrock in this part, however, is buried under thick layers of Preclassic and Classic construction fills. When we consider the volumes of soil and bedrock removed, the labor investment in the construction of the plateau was greater than the earth-fill volumes alone suggest. If the carving of plateau edges in the southern and northwestern sections occurred during the Real phase, it would make the construction efforts during this initial period even more impressive.

## Pyramid volume estimates

We also calculated the earth-fill volumes of the pyramids built on the plateau, including Structures A-3, A-5, A-6, A-10, A-20, and A-24 (Fig 3, Table A). The HP determined that Structure A-3 was constructed during the Terminal Classic Bayal phase, and our deep excavations exposed various construction layers in Structures A-10 and A-20. Internal constructions of Structures A-5, A-6, and A-24, however, have not been investigated. For the period-by-period reconstruction of the plateau, we made our best estimates for those structures, but for the volumes of the pyramids, we only calculated their total volumes.

We defined the footprint of each pyramid by examining the LiDAR data and clipped the current-ground DEM and the rectified bedrock model with those footprints. We then calculated differences between the clipped ground DEM and the clipped rectified bedrock model to obtain the volumes for the pyramids. These values may include early constructions other than pyramids, and thus may inflate the pyramid volumes beyond the real figures. These calculated volumes demonstrate our main observation that the construction volumes of pyramids were substantially smaller than those of the plateau.

## Labor investment estimates

Archaeologists have estimated construction costs through experimental studies [1-5]. Our estimates of labor investments in the construction of the Ceibal plateau were based on those earlier studies (Table 3). We made the following calculations with the understanding that most parts of the Ceibal plateau consisted of earth. For the procurement of construction materials, Erasmus noted that in a 5-hour work day a laborer dug 2.6 m^3^ of earth [1]. This provided a basis for our low estimates of labor investment. Gomez-Pompa et al. gave a lower figure of 1.1 m^3^, which led to our high estimates [6]. For the transport of materials, we assumed that a substantial part of the earth used for the Ceibal plateau was obtained from poorly-drained areas around Group A, and builders transported the materials over an average distance of 500 m. Erasmus stated that his worker carried 500 kg or 0.384 m^3^ of material a day for this distance, which resulted in our low estimates. Abrams used the formula of Q x [1/(L/V + L/V’)] x H, where Q is the load for a single trip, L is the transport distance, V is the velocity with a load, V’ is the velocity without a load, and H is the work hour per day [2]. He used a value of 22 kg for Q, 3 km/h for V, 5 km/h for V’, and 5 hours for H. This formula yielded a figure of 412.5 kg or 0.316 m^3^, providing a basis for our high estimates.

For fill construction, Abrams suggested that most fill materials at Copan, where he conducted his study, were simply thrown in, and this labor investmen was subsumed in transportation costs. Following his assumption, our low estimates did not include fill construction costs. Nonetheless, most fills of the Ceibal plateau were carefully prepared, involving the construction of division walls and thorough compacting. Abrams proposed a figure of 4.8 m^3^ per person-day for the construction of elaborate fills, which was used for our high estimates.

Although the Ceibal plateau was made mostly of earth, it also contained some stone retainig walls. In addition, varying numbers of masonry structures stood on the plateau during different periods. Although we do not have sufficient data to assess the quantities of these masonry constructions, it is desireable to take these constructions into account. Studies at other sites may provide points of reference. Webster and Kirker, for example, estimated the construction volume of Tikal Temple 1 at 18,260 m^3^ and its labor input 90,000 person-days [4]. The formula of our high estime indicates that a building of the same volume made entirely of earth would require 78,102 person-days, a cost 15 % lower than that of Tikal Temple 1. We feel that Webster and Kirker may have substantially underestimated the construction effort of this high pyramidal building. Nonetheless, rates of labor investment at Ceibal should not be greater than that of Tikal Temple 1 because most Ceibal masonry buildings were less elaborate. We used the following arbitrary figures to adjust our high estimates to account for stone construction materials. For the Real phase, when the use of stone was uncommon, we assumed that the total labor investment in the plateau construction including masonry was 2 % greater than the estimate for a construction consisting only of earth. As stone constructions became more common over time, we applied adjustment rates of 5 % for the Escoba, 8 % for the Cantutse-Xate, and 10 % for the Classic period.

# References

1. Erasmus CJ. Monument building: some field experiments. Southwestern Journal of Anthropology 1965;21:277-301.

2. Abrams EM. How the Maya built their world: energetics and ancient architecture. Austin: University of Texas Press; 1994.

3. Abrams EM. Economic specialization and construction personnel in Classic period Copan, Honduras. American Antiquity 1987;52(3):485-499.

4. Webster D, Kirker J. Too many Maya, too few buildings: investigating construction potential at Copán, Honduras. Journal of Anthropological Research 1995;51(4):363-387.

5. Ortmann AL, Kidder TR. Building Mound A at Poverty Point, Louisiana: Monumental Public Architecture, Ritual Practice, and Implications for Hunter‐Gatherer Complexity. Geoarchaeology 2013;28(1):66-86.

6. Gomez-Pompa A, Morales HL, Avilla EJ, Avilla JJ. Experiences in traditional hydraulic agriculture. In: Flannery K, editor. Maya subsistence: studies in memory of Dennis E. Puleston London: Academic Press; 1982. p. 327-342.


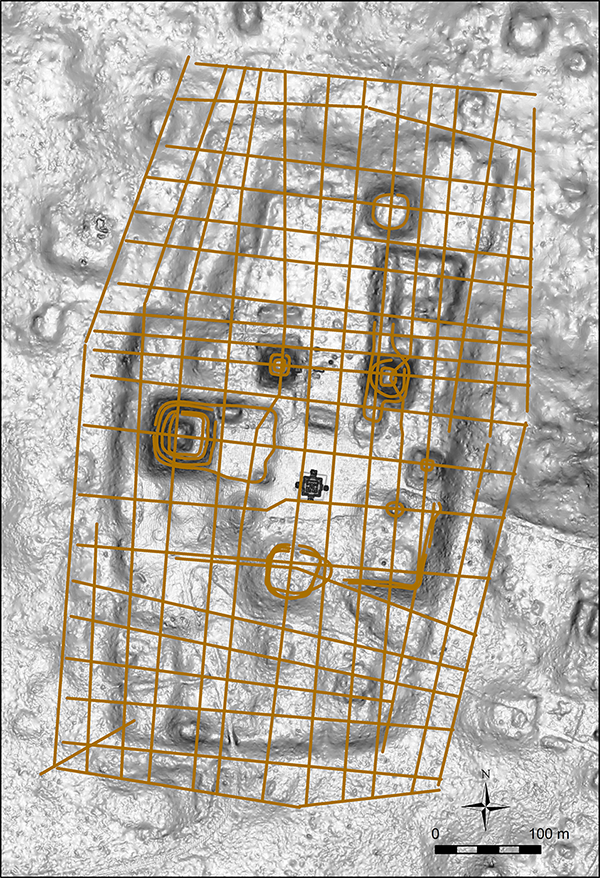


Fig A. LiDAR-derived DEM of the Ceibal plateau with the lines of sections and contours used for the creation of 3D digital models of the plateau by period.

Table A. Estimated volumes of the plateau and pyramids.

|  | | Volume | Yearly | % of |
| --- | --- | --- | --- | --- |
|  | | (m^3^) | average† | pyramids |
| Plateau* | | | | |
|  | Minimum | 587,121 | 301 | 12.9% |
|  | Most plausible | 711,906 | 365 | 10.6% |
|  | Maximum | 892,055 | 457 | 8.5% |
| Pyramids | |  |  |  |
|  | A-3 | 1,270 |  |  |
|  | A-5 | 1,506 |  |  |
|  | A-6 | 4,037 |  |  |
|  | A-10 | 24,741 |  |  |
|  | A-20 | 13,304 |  |  |
|  | A-24 | 30,648 |  |  |
|  | Pyramid total | 75,506 | 39 |  |

*The volumes of the plateau include those of pyramids and other structures standing on it.

†Volume divided by 1950 years, that is, from the beginning of the Real phase (1000 B.C.) to the end of the Bayal phase (A.D. 950).

Table B. Plateau volume estimates by period.

| Period | | Years |  | Max Preclassic | |  | Most plausible | |  | Min Preclassic | |
| --- | --- | --- | --- | --- | --- | --- | --- | --- | --- | --- | --- |
|  | |  |  | Volume | Yearly |  | Volume | Yearly |  | Volume | Yearly |
|  | |  |  | (m^3^) | average |  | (m^3^) | average |  | (m^3^) | average |
| Preclassic* | | 1175 |  | 564,825 | 481 |  | 550,587 | 469 |  | 425,186 | 362 |
|  | Real | 300 |  |  |  |  | 98,827 | 329 |  |  |  |
|  | Escoba | 350 |  |  |  |  | 203,040 | 580 |  |  |  |
|  | Cantutse-Xate | 525 |  |  |  |  | 248,721 | 474 |  |  |  |
| Classic* | | 775 |  | 147,081 | 190 |  | 161,319 | 208 |  | 286,720 | 370 |

*Estimates of the Preclassic and Classic fill volumes are based on the most plausible total volume of the plateau shown in Table 1.
